# Navigating Ambiguity and Uncertainty in End-of-Life Care Research Practice

**DOI:** 10.1177/15562646251377121

**Published:** 2025-09-24

**Authors:** Tom van Drimmelen, M. Nienke Slagboom, Ria Reis, Lex M. Bouter, Jenny T. van der Steen

**Affiliations:** 1 Department of Public Health and Primary Care, 4501 Leiden University Medical Center, Leiden, the Netherlands; 2 Amsterdam Institute for Global Health and Development, Amsterdam, the Netherlands; 3 The Children's Institute, 37716 University of Cape Town, Cape Town, South Africa; 4 Amsterdam Public Health research institute, Department of Epidemiology and Data Science, Amsterdam University Medical Centers, Amsterdam, the Netherlands; 5 Department of Philosophy, Faculty of Humanities, Vrije Universiteit Amsterdam, Amsterdam, the Netherlands; 6 Department of Primary and Community Care, Radboud University Medical Center, Nijmegen, the Netherlands; 7 Radboudumc Alzheimer Center, Nijmegen, the Netherlands

**Keywords:** researcher discretion, ethnographic research, researcher degrees of freedom, responsible conduct of research, uncertainty, moral ambiguity, end-of-life care research

## Abstract

Researchers frequently face conceptual, methodological, and ethical decisions — collectively known as researcher discretion — that can substantially influence research outcomes. Despite its importance, the nature of these discretionary moments and their triggers in everyday research practice remain underexplored. Based on an abductive thematic analysis of fieldnotes, documents, and interviews from twelve months of ethnographic fieldwork in two end-of-life care research groups between 2020 and 2022, we found that uncertainty and moral ambiguity prompted researchers to shift from intuitive to reflective decision-making, requiring discretion. While uncertainty could in principle be reduced by further inquiry, practical constraints often made this unworkable. Moral ambiguity stemmed from conflicting values that no additional information could resolve. These findings suggest that strict adherence to protocols or ethical frameworks may be insufficient to ensure responsible conduct of research, and should be complemented by building researchers’ capacity to navigate discretion and both identify and responsibly exercise their researcher discretion.

## Introduction

Throughout the process of planning, conducting, and reporting research, a researcher will encounter conceptual, methodological, and ethical decisions to make, amounting to their researcher discretion. This discretion is referred to by many different names. “Researcher initiative” (Glaeser, 2006), “researcher latitude” (Fletcher & Black, 2007; Humphreys et al., 2013; Mayo, 2018), or “researcher degrees of freedom” (Simmons et al., 2011), all refer to the substantial discretion that researchers face in conducting their research.

Situations where individual researchers confront difficult questions with ambiguous outcomes has long been a topic of researchers of science and technology studies, mostly built on the foundations constructed by authors such as Knorr Cetina (1981, 1999); Latour (1987); Latour and Woolgar (1979), and Lynch (1985). More recently, following the discovery that the reproducibility of research leaves much to be desired (Begley & Ellis, 2012; Cobey et al., 2023; Ioannidis, 2005; Open Science Collaboration, 2015), research conduct is quickly unfolding to be a major topic in other disciplines as well (Aubert Bonn & Pinxten, 2019; Hiney, 2015).

Research on this discretionary space is important considering the extensive effect that researcher discretion could have on outcomes of a study. To facilitate and guide researchers to exercise their discretion responsibly, we need to understand what these decisions consist of. But before we can analyse the specific considerations and heuristics that researchers may use to come to their decisions, we first need to explore what researchers see as a decision (van Drimmelen et al., 2024). American pragmatist philosopher John Dewey wrote that “*[t]hinking is not a case of spontaneous combustion. (…) There is something specific which occasions and evokes it”* (Dewey, 1910, p. 12). In this article, we will describe what occasions and evokes discretion in research practice.

In doing so, we aimed to look further than the ex-post accounts that provide a sanitised version of the practice of research. The methodology of ethnography is best equipped for this task as it allows the researcher to acknowledge and explore the inherent *messiness* in research practice and, crucially, accommodate for it in the reporting. Ethnographic methods focus on observation of people going about their everyday lives and explore the logic(s), value systems, and contexts that guide social practices (Daynes & Williams, 2018). We conducted this ethnographic research in end-of-life care research groups, which itself well for research on researcher discretion as it may be particularly visible due to its emotionally charged subject. In addition, randomised controlled trials or other established study designs that are supposed to limit researcher discretion best are rarely feasible or ethically acceptable in end-of-life care research (Grande & Todd, 2000).

## Methods

This study is part of a larger ethnographic research project on researcher discretion, and builds upon our earlier report on the identification of instances of researcher discretion (van Drimmelen et al., 2024). Our project protocol was pre-registered on the Open Science Framework (https://osf.io/qmdh5). The project was deemed not subject to the Dutch Medical Research with Human Subjects Law by the Medical Ethics Review Board Leiden, The Hague, Delft (23-09-2020, #N20.131). Informal methodological advice was provided by the Amsterdam Institute of Social Science Research (AISSR) Ethics Advisory Board.

### Data Collection

A combination of participant observation, document analysis and semi-structured interviews allowed us to observe the everyday decision-making as it happened, and to describe the deliberative processes and the context of researcher discretion in daily practice. The first author (TvD) conducted fieldwork at two end-of-life care research groups in North-West Europe between November 2020 and February 2022.

Research groups were approached through their primary investigator, who after explanation of our research aims and methods inquired whether the other group members would be interested in participating. At least one session per group was organised during which we explained our research aims and methods to the entire research group. Though none of the participating researchers were acquainted with the ethnographer (TvD) before the study, most of them knew the principal investigator (JvdS) either from her work, or as a collaborator. This proved conducive in gaining the trust required for participating in this ethnographic study. As a result, no research group decided against participation.

Because of the sensitive nature of the observations we anonymised names of individuals, institutions and locations (Iphofen, 2013; Walford, 2005). For this reason, we are unable to provide exact demographics of the participating research groups in this report. The participating research groups consisted of 15–25 persons, including senior researchers, postdoctoral researchers, PhD candidates, junior researchers, research interns, and research support staff. In choosing our fieldwork locations we sought to balance the variability and depth of the data. Therefore, the two groups that participated in our research differed in main methodology: one being more qualitatively focused, and the other using quantitative, qualitative, and mixed methods. To allow for a degree of immersion necessary for ethnographic observation, each period of fieldwork spanned six months (Creswell & Miller, 2000; Goffman, 1989). Fieldwork was scheduled to start in March 2020 but was delayed because of the Covid-19 pandemic.

During the fieldwork, the first author was embedded fulltime in the participating research groups; he was present during regular working hours, attended professional and social functions with the groups, and sat in on weekly project meetings. In both research groups, three research projects for detailed observation were selected by the ethnographer in consultation with the principal investigators. At the first research group, 73 meetings in total were observed; at the second research group 45 were observed. The bulk of the data consists of fieldnotes from meetings. These fieldnotes are detailed descriptions of events based on heuristic jottings made during the observation (Emerson et al., 2011). In addition, various documents, such as minutes, agendas, and manuscripts, were collected for analysis. When observational data required further explanation by the participants, the ethnographer asked for additional information directly or scheduled a more or less formal interview, which could range from a lunch meeting to a formal interview lasting between 30–75 min. For formal interviews, interview guides were drafted on the basis of specific observations concerning the individual researcher. Member checking occurred continuously throughout the research, both as a method to raise validity as well as a courtesy to the participating research group (Cho & Trent, 2006; Koelsch, 2013).

Following practice set out by Jerolmack (2013) and Livne (2019) we distinguished between two types of quotes in the findings section. We used double quotation marks to denote direct quotes that were either directly noted down or audio recorded. Single quotation marks denote an interaction reconstructed from the fieldnotes.

The stretches of fieldwork took place during different periods of the COVID-19 pandemic. The pandemic rendered traditional ethnographic research based on physical attendance at times impossible. We followed the workflows of the respective groups, which meant that fieldwork at one group took place almost entirely digitally. As a result, a considerable part of the fieldwork consisted of online (semi-)formal group meetings.

### Analysis

Atlas.ti coding software was used in the data analysis. Data analysis was performed by TvD under supervision of NS, RR, and JvdS, with whom non-recognisable excerpts were discussed.

We performed an abductive thematic analysis. This type of analysis is characterised by an iterative cycle between alternating deductive phases in which working hypotheses were formed, and inductive phases in which these hypotheses were tested; the aim of which was to search for instances which would disprove the hypotheses (Timmermans & Tavory, 2022). Practically, we roughly followed Thompson's (2022) approach for abductive thematic analysis; starting with a phase of familiarisation with the data, followed by coding, development of themes, theorising, and writing up the findings. For our previous report of this ethnographic study, we coded and analysed instances of researcher discretion. Grouping coded passages in the data that correspond to the same category of researcher discretion led to iteratively developed axial codes, or categories of researcher discretion (van Drimmelen et al., 2024). These resulting categories were subsequently analysed to come to the themes presented in this report. For the current report, we analysed the content of these instances of researcher discretion, to identify what the categories of doubt in each instance were. The findings in the current article result from this analysis.

### Informed Consent, Confidentiality, and Data Management

All participating researchers signed an informed consent sheet. This consent sheet can be found on the pre-registration. However, following standard ethnographic practice, consent to participate in this research was ‘fluid’; seen as a process, not an event (Iphofen, 2013). As such, the participants’ consent was renegotiated and reiterated when necessary. Participants were informed that they could withdraw or alter their consent at any point throughout the research, without having to provide a reason.

Because of the potentially sensitive nature of the observations, we chose to report the study in a way in which the participating research groups are not identifiable (Iphofen, 2013; Walford, 2005). To maximise non-recognisability, the following measures were taken. Each participant name was pseudonymised before they entered the ethnographer's notebook, digital fieldnotes, or transcripts so no real names are present in the primary data. These pseudonyms were chosen with the help of a random name generator to avoid associations to the real name. However, there may have been cases in which the specificity of the context of the case meant that we were unable to describe the decision accurately and non-recognisable at the same time. In these cases, we followed ethnographic practice and omitted, moved, or changed non-relevant contextual aspects to render the case non-recognisable in publication and presentation (Murphy et al., 2021; Saunders et al., 2015; van den Hoonaard, 2003). For example, if a person's gender was irrelevant to the research analyses, this may have been changed in this publication to maximise non-recognisability. This implies that the data provided in this publication cannot be used to serve arguments other than the ones central to this manuscript. As an additional check on non-recognisability, representatives of both participating research groups were asked to review the manuscript on recognisability of this article and deemed it sufficiently non-recognisable for academic publication.

Data is stored on a secure drive and only accessible to the first author, though non-recognisable excerpts were shared with the other authors for analysis. We acknowledge the potential value of providing an open dataset in general (Nosek et al., 2015), including in ethnographic research (Dilger et al., 2019; Murphy et al., 2021). However, because of the abundance of identifying factors in this projects’ fieldnotes, we decided in advance not to share this project's data.

## Findings

### Two Categories of Doubt

Our analysis resulted in the formulation of two types of doubt we observed, which may be described as uncertainty and moral ambiguity. Wherever researchers in our study identified discretion – a decision to be made – their considerations belonged to either one of these two categories.

The first category of considerations was marked by uncertainty, typified by a lack of sufficient information to make a reasoned decision. This uncertainty can be illustrated by researchers struggling to make a decision because of inability to judge the burden to a particular patient, to ascertain the meaning of a participants’ answer, or which strategy will lead to a better recruitment rate. However, we observed that a lack of information was not the only source of doubt. Considerations that fell under moral ambiguity were characterised by the relevance of two or more distinct values that were either incompatible and/or incommensurable with one another. For example, the value of openness could prescribe an action that conflicted with the value of privacy of a participant, or – as discussed in detail below – the value of research quality could clash with the value of care for the participant. Both categories are described in more detail below.

### Uncertainty

In the research processes that we followed we observed extensive uncertainty. For example, uncertainty occurred about what method of analysis would serve best, whether an error had been made in the coding process, whether a participant was able to give informed consent or sufficiently mastered the language of interviewing, or whether a researcher was skilled enough for a particular task. What connects all these decisions is that the researchers lacked the necessary information to make a reasoned decision. They faced uncertainty, and thus the decision was left to their judgment, their discretion.

During the analysis, we came to the insight that it would be valuable to distinguish between two types of uncertainty in our data: concrete and interpretational. First, concrete uncertainty occurred when some material value – e.g., a quantity – was simply unknown. For example, researchers may not know how many eligible participants will be available at a particular inclusion site, or how many months a review of their manuscript may take. Second, interpretational uncertainty involved all decisions related to meaning, for example dealing with a concept that was difficult to translate or choosing the most suitable method of analysis. As a quick introduction, the reader will find a Table 1 below containing examples of either type of uncertainty that emerged during our observations.

**Table 1.** Examples of Instances of Doubt Arising from Uncertainty Observed During Fieldwork.

Uncertainty	
Concrete uncertainty	Interpretational uncertainty
How many eligible participants will be available at an inclusion site?	How can we best translate a concept
How long will a particular type of analysis take?	How do we best interpret earlier research plans
How long will it take to fix a bug in the data collection software?	What is the best method of analysis?
Will the study be finished by the time of a particular conference?	Whether a (student) intern is suitable and capable enough for a part of the research execution
How many participants will we be able to include before the deadline?	What is the right authorship order: How to weigh contributions to a research project?
How long will a research team member remain on leave (parental/illness)?	Does the research participant sufficiently master the language of the study?

#### Concrete Uncertainty

Most of the individual research projects that we observed faced some form of problems with recruitment. And how this was to be remedied was usually unclear. For an example of this situation, we find ourselves in a project meeting with three researchers: PhD researcher Vera, and her two supervisors Lydia and Lisa. The three of them are slowly becoming lightly exasperated. For weeks now they have been discussing the same topic; the lagging recruitment rates. They are looking at an excel sheet containing all information about the recruitment of this project: e.g., the date of first contact, date of consent to participate, by what means the participant was recruited, etc. “As you can see, things aren't going quite the way I want them to yet,” Vera says. She explains that she sent out newsletters to recruiting partners and asked all current participants whether they knew of any eligible participants as well; “But I don't know what else I can do at this point”. Ideas on how to improve the recruitment rate are abundant; ranging from flyers at healthcare practices, advertisements in local newspapers, advertisements on Facebook, to contacting previous participants whether they knew any potential new participants. However, in order to make a reasoned decision about where to divert their efforts to, the bare minimum of information necessary would be the (1) expected inclusion rate at the current inclusion sites, (2) the expected additional inclusion rate per potential new inclusion strategy, (3) the amount of resources needed per new recruitment strategy, and, lastly, (4) how these newly recruited participants would compare to their existing study population. This information is just not available to the researchers. These points of information also serves as an illustration in distinguishing concrete from interpretational uncertainty. We can see that numbers (1) through (3) are missing numbers, but point (4) is of a different nature, this uncertainty concerns a comparison of content; this uncertainty is interpretational.

#### Interpretational Uncertainty

During the fieldwork we observed various instances in which researchers were uncertain about how to interpret something. For example, we observed many instances in which the researchers were unclear what sentiment a participant was exactly expressing. In our data, this uncertainty could stem from a participant's use of a concept that was difficult to translate, or an answer that allowed for two or more different interpretations, or one that was conditional. For an example of the latter, we can listen to Emma, a PhD researcher, who is describing to her supervisors a difficulty she has encountered in conducting interviews. She explains that ‘there's one question that many participants find tricky’, and notes that this question asks about a patient's approval of a medical professional administering a particular intervention. Emma says that she found that generally, ‘participants indicated that if it were done in consultation [with them] they would agree, but if it were done unilaterally by the doctors they would not;’ “which box do I tick then?”. This interpretational uncertainty concerning interpretation did not just emerge between the researchers and participants, but also between researchers themselves. In fact, our observations also show frequent instances of this type of uncertainty arising within a single researcher, who expressed uncertainty about something they themselves had written down earlier: “yeah actually I don’t know anymore, two-three years ago … What clever thing did I come up with?”.

During our ethnographic fieldwork, discretion often emerged from uncertainty. However, uncertainty was not the only category of doubt in research practice that we encountered.

### Moral Ambiguity

This category of doubt emerged when researchers faced multiple potentially incompatible and/or incommensurable values. Following all relevant values in research practice occasionally proved to be difficult for the researchers we observed. Examples of these values that potentially conflicted in our observations include transparency, feasibility, completeness, timeliness, and care for the participant. For examples of practical situations of doubt arising from moral ambiguity we direct the reader to (Table 2).

**Table 2.** Examples of Instances of Doubt Arising from Moral Ambiguity Observed During Fieldwork.

Moral ambiguity
How much participant burden is justified for the sake of research?
Does a situation warrant the violation of honesty towards participants if this honesty may bias or invalidate the research results?
Does a situation warrant the violation of honesty towards funders?
Does a situation warrant working outside of working hours?
Does a situation warrant the violation of a local law, or professional regulation?

An in-depth analysis of all relevant values is beyond the scope of this particular report, though certain values are discussed more in greater detail below. Specifically, we describe the occasionally incompatible and/or incommensurable values of methodological rigour and care for the participant, as this was one of the most common and exemplary cases of moral ambiguity.

#### Case Study: Methodological Rigour and Care for the Participant

In its simplest form, the researchers were required to weigh the burden to a particular patient, or group of patients, against the expected benefit of the improved care that was expected following the study's results. Importantly, because of the inherently short remaining life expectancy of the study population in end-of-life care research, those who are burdened by their participation in these studies are not likely to personally reap the benefits of improved care as a result of the study. This underscored that every inclusion in the observed studies required a careful navigation of these conflicting values.

On numerous occasions, researchers reported to their colleagues that they had struggled to determine whether the burden on a particular participant was excessive or not. Whether or not the data collection procedures were piloted or not, the heterogeneity in the study population meant that what could be an enjoyable conversation for one participant may be a challenging ordeal for the next one: either physically or mentally. One such instance concerning a progress meeting between a junior researcher of a qualitative research study and her supervisor is particularly illustrative:

Vignette 1: research progress meeting

**Table:** 

In one of the research meetings Raisa, a PhD researcher, notes to Marianne, her supervisor, that she experienced some difficulty with potentially overburdening their research participants. She explains that upon asking particular questions, certain participants “immediately start crying (…) it moves them, these questions”. Marianne acknowledges this effect on the participants and reiterates that by asking these questions they are assuming a moral responsibility themselves to analyse the participants’ answers, and, specifically, a responsibility to make sure that they do not ask too much. Raisa agrees and explains that in one interview she did in fact refrain from asking about a particular topic: “you really unsettle them with such a question. (…) I didn’t ask her [about it]. (…) Because then I’m affecting her in a way that I really don’t want to …”. In response, Marianne explains that indeed no-one else but the interviewer can decide in the moment whether a question can be asked, or whether an interview should be ceased: “Only you can [decide that], you are in the moment and have to sense what is possible and what is not”. She also explicitly mentions the downside to the research quality or depth, when she notes that ‘there are always things of which we think that we really would have liked to know, but that we simply cannot touch for now’. She does, however, note that there are ways of dealing with this, as she says that “the only thing that I can give you are tricks”. These tricks included depersonalising the question, carefully leading up to the question, or framing it in a positive rather than a negative sense.

What makes the value of care for the participant so exemplary of the complexity of moral ambiguity in research practice, is that it does not only consist of managing the burden for the participants. In fact, the opposite also appeared in our observations. In these situations, the researchers noted that they perceived that participation in the research had a direct beneficial effect on the participants. This perceived benefit for participants emerged either simply because of the extra attention and interest they received during the data collection, or because of the fact that participation aided them in grappling with their anxiety concerning their impending death. This perception was strengthened by the fact that the researchers regularly encountered potential participants who actively sought to participate in different research projects. For an example of this situation we have to go back to Vera, Lydia, and Lisa: Vera explains that she received a request to participate in the research. Considering their budding exasperation about the lagging recruitment rate, this first comes as a pleasant surprise. As Vera continues, however, she explains that including this person would carry a risk of biasing their results, because the reason that this person wants to participate, is because she already has considerable knowledge of the intervention they are studying. Where Lisa seems open to the possibility, Lydia is sceptical: ‘I understand that you want to help this person, but we also have to see if you can justify it methodologically’. Ultimately, they decide this is not possible, and they will have to reject the person for the study, with Vera concluding that: “as much as I would like to help her … it is also important for the research (quality)”. In these cases, the values of methodological rigour and care for the participant were still in conflict, but inversely so. Instead of assessing whether the burden to the participant was morally warranted, the researchers needed to assess whether the inclusion of these participants actually contributed to their ability of answering their research questions.

This moral ambiguity occurred both on the individual level: ‘can we include *this particular patient*?’ – as described above – and on the systematic level: ‘can we include *patients with this particular condition*?’. For an example of this moral ambiguity on a systematic level, we find ourselves in a fully digital research meeting, where Louise, Irma, and Marianne, a junior researcher, postdoctoral researcher, and senior researcher, respectively, are discussing a project. The atmosphere is slightly gloomy, as they have a difficult decision ahead of them. Irma notes that they have not been able to recruit sufficient participants for one of the arms of their interview study, and implies that they must decide whether to try to find a way of increasing the recruitment, or to cut their losses and discontinue this particular arm of their study. As the discussion continues, care for the participants emerges as a key factor. In previously observed meetings, Marianne consistently expressed care not to burden their participants any more than is strictly necessary. They discuss the possibility that they will be able to recruit some more participants – but not enough to be able to formulate sound conclusions, in which case the participants’ burden would be wasted. Marianne notes that: “If you interview three patients then you’ve got nothing. And then you did burden these three patients”. Ultimately, the researchers concluded that cutting this part of the study was not necessary at that moment, but would be if they could not find participants soon.

The above situation also serves as an illustration of how uncertainty and moral ambiguity converge. If more information was clear about the expected recruitment rates, the discussion would be better informed. Though it would still have a moral component. Crucially, these researchers lacked the information of whether they would be able to include sufficient participants, adding an extra dimension to their doubt.

At times, the two categories of uncertainty and moral ambiguity blended together in a particular decision. For example, in the practical decision of whether to include a particular participant, the researchers in our study were first required to decide how much burden is acceptable for the participant, but they must also deal with the uncertainty resulting from the inability to accurately assess the burden to these participants. However, a crucial difference between the two categories is that doubt arising from mere uncertainty can, at least in theory, be resolved by gathering more information. Even if the information may be far too costly to obtain relative to the stakes of the decision, in theory it is possible. In contrast, doubt arising from moral ambiguity does not allow itself to be resolved by more information, as it requires a judgment of values.

### Navigating Uncertainty and Moral Ambiguity

#### Reducing Uncertainty

When faced with uncertainty, but still wanting to make a reasoned decision, we observed that the researchers engage in different uncertainty reduction strategies. One of these strategies was to consult a colleague, an expert, or the literature. For example, in one of the research projects we followed, a senior researcher, Lydia, explains to her colleagues that a research group at a different institute shared their recruitment strategies with her: “to try to keep track of what works, and what doesn’t”. For example, she says: “there are a number of closed Facebook groups that do well. And a couple of patient associations”. Or, if the study in question was conducted simultaneously at different institutions, the researchers analysed which institutions performed well on recruitment to assess their methods: “How are the Germans able to recruit so well?^1^”. Usually however, this information seemed either difficult to find, or poorly transferable to the situation at hand. Transferability of recruitment strategies was limited further by the Covid-19 pandemic, which rendered many previously successful recruitment strategies ineffective.

When the available information could not reduce the uncertainty sufficiently, the researchers occasionally engaged in creating their own information by conducting in more or less systematic testing in order to avoid an arbitrary decision. In one such case, when the researchers were faced with low recruitment rates, they probed various strategies with small efforts or funds to assess their likely costs and effects on the recruitment rate. In fact, the researchers we observed occasionally noted that it might be fruitful to attempt to set up a whole new scientific experiment on how to recruit best in the end-of-life care research discipline. However, despite all their efforts to reduce the uncertainty surrounding their decisions, the researchers were often still faced with considerable residual uncertainty, hampering their ability to make a reasoned decision. In these situations, the researchers were left to exercise their discretion as to how to continue.

#### Dealing with Moral Ambiguity

To a certain extent, the researchers’ efforts in dealing with moral ambiguity was similar to their reaction to uncertainty; either they engaged external help, or they conducted their own investigations. An example of the former is given by Marianne, who was faced with moral ambiguity regarding who could access the raw data of their project explained her thought process: “I do two things. I call the legal department, and sometimes I call an ethicist’. In cases this expert help was not deemed possible or necessary, we observed researchers manage these moral ambiguities through reflexivity. As such, we observed various instances where researchers engaged in reflexive conversations on the topic of assessing and managing participant burden. For example, in an update on the inclusion at a particular recruitment site, junior researcher Emma notes some discomfort in asking patients to volunteer their time for a survey. She says: “It does feel like you’re bothering them”. After a pause of a few moments, she adds: but that's presuming [their experience]”. In another meeting Lydia phrases their main concern: “We have to be careful that we don’t start thinking for them [the participants]”. On the other hand, the researchers also often shared experiences from they felt like their inclusion of the participant actually helped them. Minutes later in the same meeting, Emma shares an anecdote about one of her research participants who told her he ‘really enjoyed’ the participation, mostly because it allowed him to have a cigarette break. Maartje, another junior researcher agrees with Emma's conclusion, and notes: “you do offer something [to the potential participant]”.

## Discussion

In this ethnographic study of end-of-life care research, we explored how researchers navigate decision-making moments marked by two distinct but often overlapping categories of doubt: uncertainty and moral ambiguity. While uncertainty arises from incomplete or ambiguous information, moral ambiguity emerges when conflicting values — such as methodological rigour and care for the participant — prevent researchers from identifying a singular “good” action. We found that these two sources of doubt prompt a shift from intuitive, automatic judgments to more deliberate, reflective decision-making; requiring researchers to exercise discretion.

This observed shift from intuitive to reflective action connects to established theories of human decision-making, which distinguish between implicit and explicit reasoning strategies. Various authors have given equally various names to both these strategies. Examples are Kahneman and Tversky's *system 1* and *system 2* thinking (Kahneman, 2011); Posner and Snyder's *automatic* and *controlled* thinking (Posner & Snyder, 1975), but also William James’ *associative* and *true* reasoning (James, 1890). The common denominator in these theories is that most of actors’ decisions are taken fast, intuitively, and automatically, whereas some are slowly, consciously, and deliberated. By unpacking this shift through observations of end-of-life care research, we offer an insight into research practice when formal protocols or textbook moral reasoning fail to provide a clear course of action.

### Navigating Uncertainty and Moral Ambiguity in Research Practice

When faced with uncertainty, the researchers in our observations generally sought to reduce the uncertainty they faced in their decisions, as much as necessary to alleviate their doubt and to come to a decision. Interestingly, these investigative probes can be seen as a form of research-within-research, where researchers apply scientific methods to resolve problems arising during the execution of their own research plans. Importantly though, almost invariably, the amount of information gained by the researchers was not enough to determine which option should be chosen. This finding mirrors a classical view on uncertainty and human action formulated most notably by Herbert Simon (1947, 1955, 1979), who claimed that decision makers have “neither the senses nor the wits to discover an ‘optimal’ path” (1956, p. 136) and thus must find a *satisfactory* path instead. This insight has become core to economic and organisational science since, though its implications on the processes of science itself have remained underdeveloped. Though much has been written on bias in science, where it comes from (Fanelli, 2010; van der Steen et al., 2019), its impact on scientific process (Open Science Collaboration, 2015), and how to reduce bias in research practice (Baldwin et al., 2022; DeCoster et al., 2015; Nuzzo, 2015), we have yet to start the discussion on what a *satisfactory* level of bias could look like.

Beyond uncertainty, we also observed that researchers constantly had to navigate a complex constellation of values and norms – e.g., the responsible management of public funds, maximum objectivity of research, and care for the participant – making for complex and ambiguous decisions. Importantly, this is likely not merely an instance of certain researchers not being able to properly interpret the values and norms underlying these products. This was noted by Peels et al. (2019), who performed a comparative analysis of various scientific codes of conduct and concluded that there is an “irreducible value pluralism in research integrity” (p. 2). This seems to be a widely shared conclusion, as Derksen (2019) writes that “[r]ules do not determine their own application. Following a rule always requires an interpretation of what the rule means in this particular situation. In science, the gap between rules and action is particularly wide because at the forefront of research, novel situations are being created: new techniques, new instruments, and of course new phenomena and effects, which all raise the question of how the rules apply in these novel circumstances” (2019, p. 450). As an example, Derksen pointed to Karl Popper's foundational concept of falsification and points out that what constitutes falsification of any theory is still a matter of interpretation. This ongoing interpretative process underscores the inherent complexity in applying broad scientific principles to concrete research situations.

In sum, while uncertainty may sometimes be reduced by gathering additional information, moral ambiguity presents a fundamentally different challenge. Any doubt arising from uncertainty can – in theory at least – be resolved by obtaining more information. This meant that the researchers were merely, though decidedly, limited by their resources and ability to gather more information. In contrast, where moral values were involved, no amount of additional information would eliminate the doubt, as a moral judgment was required. Taken together, these findings highlight that research quality is not simply a matter of following fixed rules, but requires continuous interpretation and the exercise discretion. This leads us to our central finding: that in research practice, moments of doubt emerging from either uncertainty and/or moral ambiguity compel researchers to exercise discretion beyond formal protocols, revealing the interpretive work embedded in scientific practice.

### Strengths and Limitations

#### Note on the Scope and Aims of Our Research

It is important to note that in this study report we did not set out to describe the mental heuristics, biases, or the effects of power dynamics in the researchers’ decision making. Instead, we focused on how researchers identify and navigate their own discretion—specifically, what distinguishes implicit from explicit decisions, and what prompts researchers to recognise the need for active decision-making. As our approach is based on extended ethnographic fieldwork in two research groups in a narrow disciplinary field, we recognise that readers may have concerns about the generalisability of our findings. Our aim, however, is not to produce generalisable claims but rather to provide in-depth, situated insights that reveal challenges in research practice that may be overlooked by other methodologies.

To cross-validate emerging patterns and enhance trustworthiness of our findings—as qualitative research prioritises credibility over generalisability—we employed the following three triangulation techniques throughout the study (Denzin, 1978): 1) data triangulation: conducting ethnographic research in two distinct research groups; 2) methodological triangulation: combining participant observation with formal and informal interviews throughout the study, and; 3) investigator triangulation: weekly meetings with supervisors to discuss and reflect on field notes and interview data.

#### Note on the Validity of Ethnographic Findings

A frequently heard source of doubt on the validity of ethnographic fieldwork is that the ethnographer's presence at the participating group will cause the participants to behave differently than they would normally. The reasoning goes that participants will self-censor and present themselves in a more favourable light when they know that they are being observed. Given the extended period of fieldwork at each participating research group (six months), it is unlikely that any participant would have been able to substantially alter their behaviour for the full period of fieldwork. However, these possible observer effects were monitored throughout the study, and the ethnographer constantly reflected on his position in the group. In addition, at the end of the fieldwork periods, the ethnographer took care to reflect on his influence on the research practice with the participating researchers. In doing so, none of the participating researchers noted that their behaviour changed on the basis of the ethnographer's presence. As one of the participating researchers noted:

*“Uhm well uhm, I don't think so actually. I think that – speaking for myself – yes, that occasionally, I'm a little more aware of it and how it goes … and what you're talking about … and that you’re saying something, and why you’re saying it and so on. But I don't believe that I, that I've said different things or made different decisions or started thinking differently about making decisions or so on [because of the ethnographer's presence]”* (Interview with senior researcher).

Interestingly, this researcher, and others with her, did mention that they noticed that they were more reflective in the presence of the ethnographer, also noting that they found themselves explaining thoughts that they would have otherwise not vocalised. In this way, the visibility of the ethnographer may have even enriched the data, given that participants may have been induced to voice their considerations more (Monahan & Fisher, 2010). However, this certainly did not apply to all participating researchers, like this junior researcher, who replied that:

*“[actually] I think I've also completely forgotten what you were researching … I know you're sitting there taking notes, but I completely forgot what the research question is – by the way – or what your research questions were … So now you know that too”* (Interview with junior researcher).

We have to take into account the possibility that participants consistently painted a picture of their considerations they deemed more socially desirable than what was actually going on in their minds. Either consciously or subconsciously. Again, this possibility was worded well by one of the senior researchers.

*“Part of the scientific integrity takes place in the work meetings that you’ve observed … and part of course also in what you write down … the choices that you make in the data analysis without discussing them extensively. So yeah. Perhaps especially that process of writing down everything that you do, and everything that comes out. That is … of course you miss that if you’re only observing”* (Interview with senior researcher).

Studying this discretionary space is difficult. Large parts of these decision-making processes are often not observable, as they take place in the minds of the researchers themselves, and may indeed develop over multiple days or weeks, not simply restricted to working hours. In any case they extend beyond the observational capacity of the ethnographer. In the interviews and clarifying questions we attempted to gauge the developments outside of the ethnographer's observation. Though of course this data is of a different, secondary, nature compared to the direct observational data. Ultimately, our aim was to map and analyse a process that is impossible to observe in full, and – though complying with prevailing quality criteria for qualitative research (Frambach et al., 2013) – our study must be understood as a necessarily incomplete picture.

## Conclusion

Dewey concluded that “[t]hinking is not a case of spontaneous combustion. (…) There is something specific which occasions and evokes it” (Dewey, 1910, p. 12). Our findings indicate that that which occasioned and evoked consideration by researchers in our observations, could be categorised as either uncertainty or moral ambiguity.

These findings underline the need to acknowledge researchers’ active role in shaping the conduct of research. Rather than being able to *follow* fixed procedures, we observed that researchers often had to exercise their own discretion in uncertain or morally ambiguous situations. It is therefore essential that they are supported in developing the skills to recognise and respond to such instances. While we recognise the value of checklists, standard operating procedures, and guidelines, our findings suggest that these cannot prevent all uncertain or morally ambiguous situations. We argue that rather than offering fixed solutions to various problems researchers may face, researchers need both training and opportunities for reflection to allow them to take responsibility for the choices that cannot be prescribed.

### Best Practices

Ultimately, the presence of either uncertainty or moral ambiguity implies that research practice requires the practical judgment of individual researchers, and that focusing on cultivating this ability is an important factor in educating responsible conduct of research. Though from our empirical work we cannot make any conclusions about the possibility of eliminating this uncertainty and moral ambiguity altogether, logically we see enough reason to conclude that this is likely impossible in an absolute sense: that these two factors are an endemic part of the research process. De Vries et al. (2006) arrived at this conclusion after conducting a series of focus groups with academics, articulating this finding as follows: “The use of new research techniques and the generation of new knowledge create difficult questions about the interpretation of data, the application of rules, and proper relationships with colleagues. Like other frontiersmen and—women, scientists are forced to improvise and negotiate standards of conduct” (p. 3). In defining responsible conduct of research, it is thus imperative that this uncertainty and moral ambiguity are acknowledged.

### Research Agenda

The ethnographic methods chosen in this study allowed us to produce an in-depth insight into the practice of researcher discretion, but does not allow for inferences of generalisation. To further understand researcher discretion, it is therefore important that this study is replicated. Specifically, it is important to study the roles of uncertainty and moral ambiguity in research practice in disciplines other than end-of-life care research, to analyse whether and how disciplines differ, and which factors make up this difference. In addition, more empirical work is needed to gauge the exact depth of the uncertainty and moral ambiguity in research practice, and their cumulative effect on research outcomes.

### Educational Implications

Our findings seem to resonate with more general work on the application of norms. For example, in her attempt to explore the relationship between norms and their applications, Onora O'Neill (2007) notes that “we constantly need to act in ways that meet multiple constraints and standards” (p. 403), leading to “the substantive task of seeking ways of acting that satisfy a plurality of norms” (p. 393). It is important to recognise that these values are not “plug-and-play” concepts. They must be measured, weighed against each other, and applied onto different practices. This finding would support the argument to make space for forms of reflection in/on action (Schön, 1983), such as moral case deliberation, or sense-making, as an important pillar of responsible conduct of research training (Inguaggiato et al., 2023; Kligyte et al., 2008). This could also include practices such as structured peer reflection (Jamieson et al., 2023; Olmos-Vega et al., 2023), ethical deliberation sessions (Haven et al., 2024), or mentoring that openly discusses the kinds of discretionary decisions encountered in real projects (Anderson et al., 2007; Haven et al., 2023).

If the simple knowledge of generic codes of conduct, guidelines, and norms in a particular field may not prove sufficient for the responsible conduct of research, our attention is drawn to the practice of decision-making and researcher discretion in research projects. Therefore, we propose that efforts to improve the responsible conduct of researchers should also include building researchers’ capacity to engage with these decisions critically and proactively.

## Notes

1. Country name fictionalised for anonymity.
